# Evaluation of incidence and histolopathological findings of soft tissue sarcomas in genitourinary tract: Uludag university experience

**DOI:** 10.1590/S1677-5538.IBJU.2018.0048

**Published:** 2019

**Authors:** Berna Aytac Vuruskan, Mıne Ozsen, Burhan Coskun, Ulviye Yalcinkaya

**Affiliations:** 1 Department of Surgical Pathology, Uludag University, Faculty of Medicine, Bursa, Turkey;; 2 Department of Urology, Uludag University, Faculty of Medicine, Bursa, Turkey

**Keywords:** Genitourinary Tract Anomalies[Supplementary Concept], Sarcoma, Survival

## Abstract

**Purpose:**

In this study we aimed to review urological soft tissue sarcomas of genitourinary tract that were diagnosed in our institution and their prognostic factors for survival.

**Materials and Methods:**

The clinical and pathological records of 31 patients who had diagnosis of soft tissue sarcomas primarily originating from the genitourinary tract between 2005-2011 were reviewed.

**Results:**

The most common site was kidney (17 cases, 54.8%), and most common diagnosis was leiomyosarcoma (11 cases, 35.4%). A total of 24 patients (77.4%) had surgical excision. The surgical margins were positive in 7 patients who presented with local recurrence after primary resection. Twelve patients developed metastatic disease. During follow-up (range 9-70 month), 26 of the 31 patients (88.9%) were alive. Significant survival differences were found according to histological type (p: 0.001), with lower survival rates for malignant fibrous histiocytoma. The tumor size, the presence of metastasis at the time of diagnosis and tumor localization were not statistically significant for overall survival.

**Conclusions:**

In our series, prostate sarcomas, paratesticular rhabdomyosarcoma and malignant fibrous histiocytoma had poor prognosis, especially in patients presenting with metastatic disease.

## INTRODUCTION

Soft tissue sarcomas (STSs) are a diverse group of solid tumors that originate from embryonic mesenchymal cells which have various clinical and pathological characteristics (1). STSs of the genitourinary (GU) tract are uncommon, only accounting for 2.1% of all STSs and 1% to 2% of all malignant GU tumors (2, 3). Due to low prevalence of urological STS, clinical research is limited with institution-based studies. The tumor stage, grade, size, and localization are found to be important prognostic factors for patient survival in STSs in several studies (4-6). In this study, we aimed to review urological STS that were diagnosed in our institution and their prognostic factors for survival.

## MATERIALS AND METHODS

The clinical and pathological records of 31patients who had diagnosis of soft tissue sarcomas primarily originating from the genitourinary tract between 2005-2011 were reviewed. Sarcomas of the GU tract that arised from the kidney, ureter, bladder, prostate, and paratesticular region were included. Retroperitoneal sarcomas without a tight relationship to the kidney or to the adrenal gland were excluded. Female genital organ related sarcomas were excluded from this study. Characteristics of the patients including age, sex, tumor histology, tumor size, primary organ, metastasis at diagnosis, and status of surgical resection were examined. All histopathological findings were obtained from biopsy or surgically resected specimens from files of patients in the Department of Pathology. Local recurrence or metastasis was described as the first recurrence of disease at the primary tumor site or distant site detected by computed tomography. The clinical characteristics of the patients at the time of admission and follow-up were achieved from hospital records, directly from the patients or from their families. All patients underwent CT scan before surgery. Recurrence was defined as recurrent disease at a local or distant site.

Statistical analysis was done by using the Statistical Package for Social Sciences, ver. 13.0 for Windows, (SPSS, Chicago, IL). The crude probability of survival was estimated by using the Kaplan - Meier method and differences between patient groups were assessed by the log rank test. A P value < 0.05 was considered to be statistically significant.

## RESULTS

The demographic characteristics of the patients are presented in Table-1. The mean age at diagnosis was 58 years (range, 19-81 years). Median follow-up was 61 months. Twenty two patients were male and 9 patients were female. The most common site was kidney (17 cases, 54.8%), followed by paratesticular region with 7 cases (7 cases, 22.5%), prostate (4 cases, 12.9%) and bladder (3 cases, 9.8%). The mean tumor diameter was 14 cm. The most common diagnosis was leiomyosarcoma (11 cases, 35.4%), followed by liposarcoma (8 cases, 25.8%), malignant fibrous histiocytoma, (4 cases,12.9%), malignant nerve sheath tumor (3 cases, 9.7%), rhabdomyosarcoma (2 cases,6.5%) and Kaposi sarcoma (2 cases, 6.5%). A total of 24 patients (77.4%) out of 31 patients had surgical excision. The surgical margins were positive in 7 patients who presented with local recurrence after primary resection. Twelve patients developed metastatic disease. There were lymph node metastasis in 2 patients and distant metastasis in 10 patients; lung, liver and bones were the most affected sites. At the end of the study period (range 9-70 month), 26 of the 31 patients (88.9%) were alive.

**Table 1:** Distribution of 31 genitourinary sarcomas according to selected clinical and pathological characteristics.

Pt. No	Age (years)	Sex	Size (cm)	Location	Type	Local recurrence	Metastases	Treatment	Current status	Median follow-up (months)
1	81	M	3	Prostate	MFH	-	None	Bx	DOD	13
2	56	M	23	Bladder	LS	-	None	S	NED	66
3	42	M	5	Perirenal	MFH	+	None	S	DOD	20
4	64	M	4	Bladder	IMF	-	None	S	NED	70
5	59	M	4	Paratesticular	LS	+	Lung	S	AWD	61
6	68	M	22	Perirenal	LMS	+	Liver, bone	S	AWD	58
7	43	M	4	Paratesticular	RMS	+	Lung	S	DOD	24
8	73	M	23	Perirenal	LS	-	Adrenal	S	AWD	40
9	64	M	2	Paratesticular	LMS	-	None	S	NED	38
10	58	F	22	Perirenal	LS	-	None	S	NED	38
11	67	M	4	Perirenal	LMS	-	None	S	NED	31
12	58	F	9	Bladder	MFH	-	None	Bx	DOD	27
13	73	F	6	Kidney	MFH	-	None	S	NED	25
14	69	F	6	Kidney	LS	+	None	S	NED	24
15	19	M	5	Paratesticular	RMS	-	None	S	NED	12
16	56	F	9	Perirenal	LS	+	None	S	AWD	24
17	45	M	1	Perirenal	MPNST	-	None	Bx	DOD	12
18	67	M	4	Kidney	LMS	-	Adrenal	S	AWD	45
19	60	F	17	Perirenal	MPNST	-	Liver	S	AWD	15
20	59	M	3	Prostate	LMS	-	Lung	Bx	AWD	12
21	55	M	3	Kidney	LMS	-	None	S	NED	24
22	43	M	20	Kidney	LMS	-	Lymph node	S	AWD	12
23	64	M	8	Prostate	LMS	-	Bone	Bx	AWD	10
24	59	M	4	Prostate	LMS	+	Lung	Bx	AWD	20
25	59	M	1	Paratesticular	KS	-	None	S	NED	24
26	32	F	2	Kidney	LMS	-	None	S	NED	18
27	48	M	17	Perirenal	MPNST	-	Liver	S	AWD	11
28	35	M	2	Paratesticular	LMS	-	None	Bx	NED	19
29	69	M	1	Paratesticular	KS	-	None	S	NED	30
30	53	F	23	Kidney	LS	-	Lymph node	S	AWD	18
31	59	F	14	Perirenal	LS	-	None	S	NED	9

**AWD** = Alive with disease; **Bx** = Biopsy; **S** = Surgical; **DOD** = Dead of disease; **F** = Female; **LS** = Liposarcoma; **LMS** = Leiomyosarcoma; **IMF** = Inflammatory myofibroblastic tumor; **KS** = Kaposi sarcoma; **M** = Male; **Mo** = Month; **MFH** = Malignant fibrous histiocytoma; **MPNST** = Malignant nerve sheath tumor; **NED** = no evidence of disease; **RMS** = Rhabdomyosarcoma

**Figure 1:**
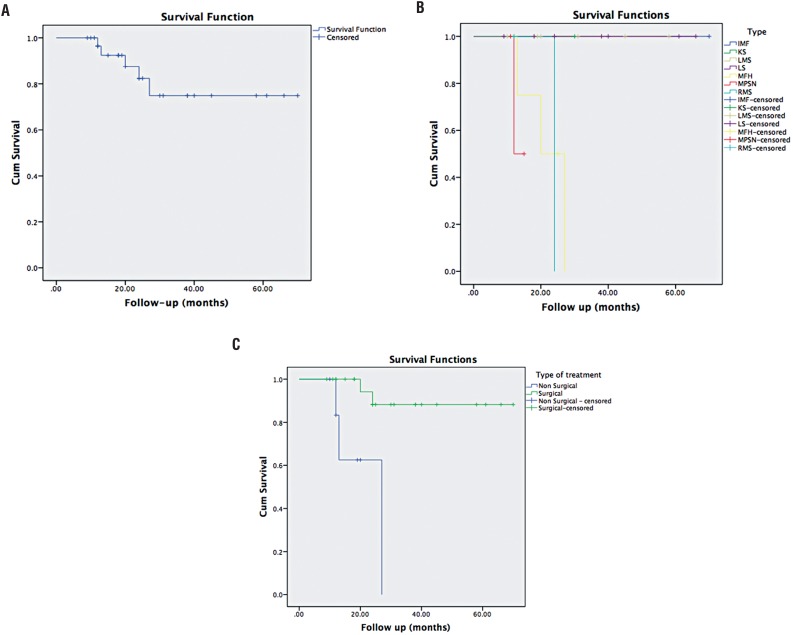
Kaplan-Meier analysis of overall survival in overall cases (A), according to histological subtype (B) according to presence vs. absence of surgical resection (C).

There were no significant differences in survival according to sex and age (p: 0.717, p: 0.107 respectively) (Figure-1A). Significant survival differences were found according to histological type (p: 0.001), with lower survival rates for malignant fibrous histiocytoma (Figure-1B). Leiomyosarcomas and liposarcomas had a better 5 years survival when compared to rhabdomyosarcomas and other types. The tumor size and the presence of metastasis at the time of diagnosis were not statistically significant for overall survival (p: 0.590, p: 0.500 respectively). There were no relation with tumor localization and survival (p: 0.449). The patients who underwent surgical resection had significantly higher rates of survival (p: 0.001), (Figure-1C).

## DISCUSSION

The infrequency of GU STSs is the main problem for clinical research while data in the literature are limited (7). In the literature, liposarcoma (LS) represents the most common soft tissue sarcoma of retroperitoneum (8). The biological behavior and prognosis of LS are usually more favorable compared to most of STS but the prognosis can vary widely depending on tumor characteristics, especially histological subtype and tumor grade (9). In our study, 6 patients had perirenal and renal LS. Renal LSs were found in renal parenchyma without being in renal lipomatous tissue. While 4 patients had no evidence of local recurrence, one patient had a local recurrence and one patient had an adrenal metastasis. One patient had a bladder LS. Primary LS of the urinary bladder is a very rare entity with a poor prognosis that usually presents as a large tumor mass (10). Our patient had no evidence of disease in 66 months follow-up. One patient had a paratesticular LS and had lung metastasis.

Leiomyosarcoma (LMS) make up 50-60% of all renal sarcomas, therefore, it is the most common histologic subtype (11). Renal LMS has a very poor prognosis, which results in death of the majority of patients within two years (12). However, radical surgery seems to offer the best chance of cure. The role of adjuvant chemotherapy and / or radiotherapy remains controversial due to the paucity of data on the treatment of this rare renal neoplasm. While local recurrence is common, low-grade LMS tends to pursue a more indolent course. Four of our patients had LMS of the kidney. All patients had no evidence of local recurrence but distant metastasis was noted in adrenal and lymph node during of follow-up. Two of our patients had perirenal LMS. Their origin has been suggested as coming from the interlacing bundles of smooth muscle in the inner layer of the renal capsule (13) and in the form of a mass attached to the cortical surface of the kidney. One of these patients had distant metastasis in liver and bone. Three of our patients had LMS of the prostate. LMS is the most common primary sarcoma of the prostate in adults and accounts for 38-52% of primary prostatic sarcomas (14). It has an aggressive clinical course (15). Due to the rarity of prostatic LMS, definite treatment recommendations are yet to be established. Multimodality treatment combinations including surgery, preoperative or postoperative radiation therapy and neoadjuvant or adjuvant chemotherapy has been used (14, 15). Up to a third of patients have demonstrable metastases at presentation, usually to the lung, and sometimes to the liver (14, 15). In our series, three patients, who had LMS, received adjuvant radiotherapy and multi-agent chemotherapy. All of them had distant metastasis in lung and bone.

Malignant fibrous histiocytoma (MFH) is rare in the urinary tract and mostly it is located in the kidneys and consists of 0.23-0.67% of all bladder tumors. In our series, the tumors were localized in kidney, prostate, and bladder. MFH usually spreads fast. The size, depth and histologic features of the tumor are important factors for metastasis (16). Despite this treatment, 3-year survival is approximately 40% (17). In our series a patient, who had MFH, underwent surgery, received adjuvant radiotherapy and multiagent chemotherapy, but died 20 months after initial diagnosis.

Two patients in our series had paratesticular rhabdomyosarcoma (RMS), which can arise from the epididymis, the mesenchymal layers surrounding the testis and its true appendages, and the spermatic cord. It is the most common primary paratesticular malignant neoplasm diagnosed in patients aged 7 years to 36 years, with a mean age of 10 years (18). With regard to pathological sub classification, these tumors were of embryonal and pleomorphic type, respectively. The patients in our series were treated by radical orchiectomy. Retroperitoneal lymphadenectomy or chemotherapy was not performed. One of our cases was found to have embryonal RMS. Although he underwent radical orchiectomy and had post-operative chemotherapy, he had distant metastasis in lung and liver. Another case had no evidence of local recurrence.

Malignant peripheral nerve sheath tumors (MPNST) are confirmed only by histological and immunohistochemically evaluation (19). Involvement of the kidney is uncommon, and it is very difficult to differentiate with other kidney sarcomas from MPNSTs clinically or upon gross examination (20, 21). Current treatment for MPNST in the genitourinary system involves surgical excision and adjuvant chemotherapy. This combined modality approach results in a 5-year survival rate of 40-69% with complete resection of localized disease (22). Two patients out of three had a poor outcome: one was dead from the disease and the other is alive with the metastatic disease.

In conclusion, genitourinary sarcomas are a rare group of tumors, some patients with localized resectable masses may have a favorable prognosis. Complete surgical resection is the best chance for survival. Prostate sarcomas, paratesticular RMS, and MFH had a poor prognosis, especially in patients presenting with metastatic disease and combined multimodality approach is the treatment of choice all of them; unfortunately, it provides limited therapeutic benefit.
